# Growth and Metal Accumulation of an *Alyssum murale* Nickel Hyperaccumulator Ecotype Co-cropped with *Alyssum montanum* and Perennial Ryegrass in Serpentine Soil

**DOI:** 10.3389/fpls.2016.00451

**Published:** 2016-04-08

**Authors:** Catherine L. Broadhurst, Rufus L. Chaney

**Affiliations:** ^1^Environmental Microbiology and Food Safety Laboratory, U.S. Department of Agriculture Agricultural Research ServiceBeltsville, MD USA; ^2^Department of Food Science and Nutrition, University of MarylandCollege Park, MD, USA; ^3^Crop Systems and Global Change Laboratory, U.S. Department of Agriculture Agricultural Research ServiceBeltsville, MD, USA

**Keywords:** *Alyssum murale*, *Lolium perenne*, nickel hyperaccumulators, ryegrass, co-cropping, phytoremediation, phytomining

## Abstract

The genus *Alyssum* (Brassicaceae) contains Ni hyperaccumulators (50), many of which can achieve 30 g kg^−1^ Ni in dry leaf. Some *Alyssum* hyperaccumulators are viable candidates for commercial Ni phytoremediation and phytomining technologies. It is not known whether these species secrete organic and/or amino acids into the rhizosphere to solubilize Ni, or can make use of such acids within the soil to facilitate uptake. It has been hypothesized that in fields with mixed plant species, mobilization of metals by phytosiderophores secreted by Graminaceae plants could affect *Alyssum* Ni, Fe, Cu, and Mn uptake. We co-cropped the Ni hyperaccumulator *Alyssum murale*, non-hyperaccumulator *A. montanum* and perennial ryegrass in a natural serpentine soil. All treatments had standard inorganic fertilization required for ryegrass growth and one treatment was compost amended. After 4 months *A. murale* leaves and stems contained 3600 mg kg^−1^ Ni which did not differ significantly with co-cropping. Overall Ni and Mn concentrations were significantly higher in *A. murale* than in *A. montanum* or *L. perenne*. Copper was not accumulated by either *Alyssum* species, but *L. perenne* accumulated up to 10 mg kg^−1^. *A. montanum* could not compete with either *A. murale* or ryegrass, and neither *Alyssum* species survived in the compost-amended soil. Co-cropping with ryegrass reduced Fe and Mn concentrations in *A. murale* but not to the extent of either increasing Ni uptake or affecting plant nutrition. The hypothesized *Alyssum* Ni accumulation in response to phytosiderophores secreted by co-cropped grass did not occur. Our data do not support increased mobilization of Mn by a phytosiderophore mechanism either, but the converse: mobilization of Mn by the *Alyssum* hyperaccumulator species significantly increased Mn levels in *L. perenne*. Tilling soil to maximize root penetration, adequate inorganic fertilization and appropriate plant densities are more important for developing efficient phytoremediation and phytomining approaches.

## Introduction

More than 400 plant species are known to naturally accumulate high levels of metals such as Cd, Cu, Co, Mn, Ni, and Zn (Baker et al., 2010; Krämer, 2010; van der Ent et al., 2013). The genus *Alyssum* (Brassicaceae) contains the greatest number of reported Ni hyperaccumulators (50), many of which can achieve 30 g kg^−1^ Ni in dry leaf biomass (Baker and Brooks, 1989; Reeves and Adigüzel, 2008; van der Ent et al., 2015). Previously we have demonstrated commercially feasible phytoremediation and phytomining technologies that can potentially clean up Ni-contaminated soils and recover high purity Ni metal (Chaney et al., 1999, 2010; Li et al., 2003a,b; Nkrumah et al., 2016). The technology employs the Ni-hyperaccumulating species *Alyssum murale* and *A. corsicum* to phytoextract Ni from a range of Ni-rich soil types. *A. murale* and *A. corsicum* are endemic to serpentine soils developed from ultramafic rock throughout Mediterranean Southern Europe.

Ni localization patterns have been determined for 10 *Alyssum* Ni hyperaccumulator species/ecotypes (Krämer et al., 1997; Psaras et al., 2000; Küpper et al., 2001; Kerkeb and Krämer, 2003; Marmiroli et al., 2004; Broadhurst et al., 2004a,b, 2009; McNear et al., 2005; Asemaneh et al., 2006; Tappero et al., 2007). Nickel is stored mainly in the leaves, and is particularly concentrated in in vacuoles of epidermal cells and trichome pedicels. *Alyssum* hyperaccumulators also accumulate appreciable Mn in the same locations that contain Ni (Broadhurst et al., 2004b, 2009).

Although Ni hyperaccumulation is a constitutive property for these *Alyssum* species, it is not known whether they secrete organic and/or amino acids into the rhizosphere to solubilize Ni, or can make use of such acids within the soil to greatly facilitate uptake. Other than rhizobiome interactions, there is essentially no evidence for unusual ligand species or highly elevated ligand concentrations associated with Ni in *Alyssum* (McNear et al., 2010; Centofanti et al., 2013). There is evidence that rhizosphere bacteria endemic to serpentine soils may stimulate Ni uptake and this may be an important factor explaining why field trials and native vegetation consistently outperform pot and hydroponic studies with respect to phytoextraction yields (Abou-Shanab et al., 2003, 2007; Rajkumar et al., 2009, 2013; Cabello-Conejo et al., 2014; Visioli et al., 2015). Two serpentine-endemic bacteria in particular (*Microbacterium arabinogalactanolyticum* and *M. oxydans*) were shown to strongly increase Ni accumulation in *A. murale* (Abou-Shanab et al., 2003, 2007). Similarly, endemic *Arthrobacter sp*. increased Ni uptake in *A. pintodasilvae* and *A. serpyllifolium* (Cabello-Conejo et al., 2014).

Cd/Zn hyperaccumulators have not shown evidence for specialized ligand secretion into the rhizosphere either (Zhao et al., 2001; Whiting et al., 2001a; Sterckeman et al., 2005; Wang et al., 2006). Specifically, root exudates collected from the Cd/Zn hyperaccumulator *Noccaea caerulescens* F.K. Mey (Brassicaceae) (syn. *Thlaspi caerulescens* J &C Presl) did not mobilize Cd, Cu, Fe, or Zn (Zhao et al., 2001). Further, Cd/Zn hyperaccumulators may not take advantage of potential phytosiderophore-related improvements in metal solubilization provided by intercropping with Graminaceae.

The grass family of plants differs from all other plant families by using a different mechanism of absorbing Fe from soils. All other species use a combination of acidification of the rhizosphere and reduction of ferric to ferrous coupled with absorption of ferrous ion. Instead, Graminaceae use a combination of chelating amino acids, the phytosiderophores of the mugineic acid family of compounds, for specific uptake of intact Fe–phytosiderophore chelates. It is known that phytosiderophores are not highly specific to Fe and can increase mobilization and possibly support uptake of Zn, Mn, Ni, Cu, and Cd as well (Zhang et al., 1991a,b; Marschner and Römheld, 1994; Awad and Römheld, 2000). Intercropping peanut (*Arachis hypogaea* L.), for example, with maize, oats, barley or wheat significantly increased Fe, Cu, and Zn uptake to the extent that Fe deficiency in peanut could be mitigated (Zuo and Zhang, 2011).

Previous results from co-cropping hyperaccumulators and grasses are mixed. *N. caerulescens* had no increase in Cd or Zn concentration when grown in the same pot with ryegrass (*Lolium perenne* L.), but yield was almost doubled in an experiment where plants were grown with sufficient time and soil volume to establish potential rhizosphere interactions with or without root mingling (Jiang et al., 2010). It was determined that the ryegrass did not solubilize Cd and Zn, however Fe was not discussed. The improved yield could be at least partially due to improved Fe availability. Increased P availability from arbuscular mycorrhizal fungi which are known to colonize Graminaceae including ryegrass (Grimold et al., 2005) is another factor which could significantly affect yield since many of the metalliferous soils that hyperaccumulators are native to are P deficient. However, co-planting the Cd/Zn hyperaccumulator *Sedum alfredii* (Hance) with ryegrass reduced both *S. alfredii* yield and Cd uptake (Wang et al., 2013). Co-planting with corn improved *S. alfredii* yield by providing shade but did not increase Cd uptake. Cd and Zn uptake by corn was unaltered by co-cropping and corn did not suffer phytotoxicity (Wu et al., 2007).

Co-cropping barley (*Hordeum vulgare* L.) and *N. caerulescens* in multiple metal-rich soils from a biosolids management facility showed little evidence for interaction between plants other than a slight increase in Cd, Cu, Ni, Zn in co-cropped pots with root interaction vs. *N. caerulescens* alone, but this probably reflected simple depletion of metals in the relatively small soil volume utilized, and not a specific phytosiderophore mechanism (Gove et al., 2002). Again, Fe was not considered in the experiment. Both Gove et al. (2002) and Jiang et al. (2010) observed an increase in ryegrass Cd but not Zn concentrations when grown with *N. caerulescens*. Similarly, Whiting et al. (2001a) showed no interaction between *N. caerulescens* and *Festuca rubra* L. with respect to Zn levels or yield.

Improved growth and reduced Zn uptake by the non-hyperaccumulator *Thlaspi arvense* L. was reported when *T. arvense* and *T. caerulescens* were grown together in pots that allowed root intermingling. Zinc salts were added to the soils at a level that was phytotoxic to *T. arvense*. Zn hyperaccumulation by *T. caerulescens* was not affected, however yield increased when root intermingling was allowed, leading to the conclusion that this system could facilitate revegetation of contaminated soils (Whiting et al., 2001b).

Herein we report a co-cropping experiment with *Alyssum* hyperaccumulator and non-hyperaccumulator species and perennial ryegrass in a natural serpentine soil. The soil is infertile and high in Ni, but is not Ni phytotoxic (Zhang et al., 2007) and supports native vegetation. Soils such as this are candidates for Ni phytomining (Chaney et al., 2010; Nkrumah et al., 2016). We tested whether ryegrass facilitates Ni, Fe, and Mn uptake by *Alyssum*, whether co-cropping with ryegrass affects *Alyssum* yield, and whether *Alyssum* hyperaccumulator and non-hyperaccumulator species can benefit from co-cropping.

## Materials and methods

### Horticulture

**Figure 1:**
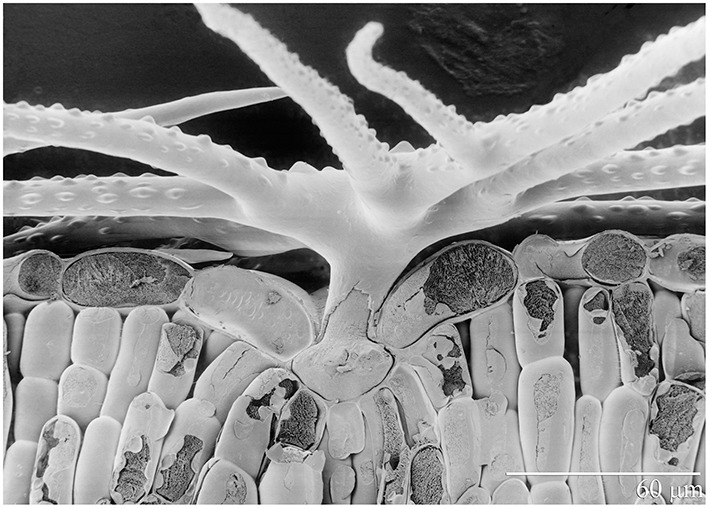
**Scanning electron photomicrograph of frozen hydrated Ni-hyperaccumulator *Alyssum murale* ‘Kotodesh’ and surface of leaf**. Note stellate trichome; Ni is very high in the vacuole below the trichome.

*A. murale* (Waldst. et Kit) “Kotodesh,” a Ni-hyperaccumulator, was grown from seed collected from a wild Albanian serpentine population. All *Alyssum* hyperaccumulator species known have leaves covered with stellate trichomes (Figure 1). Nickel is stored in leaf epidermal cells, particularly in the trichome pedicels. *A. montanum* L. “Mountain Gold” (a non-hyperaccumulator species that also has leaf trichomes) was grown from commercial seed (Hazzard's Seeds, Deford, MI). Both *Alyssum* species were started in flats with Promix® potting soil and standard fertilization (half-strength Miracle Grow®). After 40 days healthy *Alyssum* seedling roots were rinsed to remove potting medium and transplanted to prepared soils in pots. Four weeks after transplant, when seedlings had become established, commercial perennial ryegrass (*L. perenne* L. “Amazing GS,” Ampac Seed, Tangent, OR) was seeded directly into the pots. Twenty cm polyethylene pots which hold about 3 kg air dry soil were utilized. Plastic mesh was not used across the pot over the drain holes in order to avoid interference with root growth. All watering was with deionized water, and plastic trays were placed under each pot. To avoid overwatering *Alyssum*, 250–650 ml was added 2 or 3 times per week to ensure that soil dried between waterings. The co-cropped plants were grown for an additional 12 weeks.

We utilized Brockman variant serpentine soil from Josephine Co., Oregon (Typic Xerochrepts), air dried and sieved <4 mm using stainless steel sieves. Standard inorganic fertilization for serpentine soils (75 mg N as NH_4_NO_3_, 100 mg P as KH_2_PO_4_, 500 mg Ca as ${\text{CaSO}}_{4^{{}^{\circ}}}$2H_2_O, and 0.5 mg B as H_3_BO_3_ per kg soil) was added. The Brockman soil as collected is pH 6.6, very high in Ni (4710 mg kg^−1^), more than adequate in Mn and Fe, but deficient in Ca and P (Table 1). One set of treatments had 30 vol% (10%DW) aged dairy manure compost from the USDA Beltsville composting facility mixed into the serpentine soil. Fertilizer rates allowed normal growth of ryegrass on this soil which would not normally support growth of non-serpentinophytes.

**Table 1:** **Representative average Brockman soil parameters over 12 years testing**.

**Analyte**	**Units**	**Result**
pH		6.30
organic matter	%	3.1
Clay	%	21.0
Sand	%	58.0
Fe	g kg^−1^	222
Mn	g kg^−1^	3.88
Ni	g kg^−1^	4.71
Co	mg kg^−1^	354
Cu	mg kg^−1^	45.0
Zn	mg kg^−1^	180
Ca, exchangeable	meq/100 g	1.0
Mg, exchangeable	meq/100 g	6.2
Ni, exchangeable	mg kg^−1^	32.0
Ni, DPTA extractable	mg kg^−1^	124
P, Bray extractable	mg kg^−1^	0.02

*Note the very low Ca and P levels and Ca:Mg ratio characteristic of serpentine soils*.

The experiment was conducted in the USDA Beltsville greenhouse under controlled temperature and light conditions and ambient humidity. Photoperiod was 15/9 h day/night. During this time supplemental high-intensity sodium and incandescent lights capable of supplying 400 μmol m^−2^s^−1^ supplemented sunlight if necessary. Daytime temperature was 24°C with cooling initiated at 27°C. Nighttime temperature was 18°C with cooling initiated at 21°C. During the final 3 weeks of growth in late May and June supplemental lighting was turned off to avoid overheating.

### Experimental treatments

Two types of soil and six planting schemes made up 12 treatments, with three replicates per treatment. *Alyssum* plants that died soon after transplanting were replaced for the first 2 weeks of growth. Overall *Alyssum* grew 4 months after transplant, and rye grass grew 3 months after seeding. At harvest plant roots filled the pot and intermingled but plants were not pot-bound.

**Soil A**: Natural Brockman variant serpentine soil.
**Soil B:** Natural Brockman variant serpentine soil with 10 wt% compost.

**Planting Scheme**:

1. A. murale, four plants per pot spread radially and evenly.
2. A. montanum, four plants per pot spread radially and evenly.
3. *L. perenne* four groups of approximately 20 seedlings spread radially and evenly.
4. *A. murale* two plants, and *A. montanum*, 2 plants with the same plant spread as #1, alternating species.
5. *A. murale* two plants, and *L. perenne* two groups of approximately 20 seedlings, alternating species.
6. *A. montanum* two plants, and *L. perenne* two groups of approximately 20 seedlings alternating species.

### Plant material metals analysis

All clean, healthy aerial plant material was harvested. Material that was stained from the red serpentine soil or unhealthy was discarded. Harvested plant material was washed in a dilute detergent bath and rinsed in deionized water to remove adhering soil particles. Plants were dried for 72 h at 60°C, weighed, and ashed in a 480°C oven for 16 h. After cooling, the ash was digested with 2 ml concentrated HNO_3_, mixed well and then heated to dryness. The sample was then dissolved in 10 ml 3 *N* HCl, filtered through Whatman #40 filter paper and brought to volume in a 25 ml volumetric flask using 0.1 *N* HCl. Concentrations of Ca, Cd, Cu, Fe, K, Mg, Ni, Mn, P, and Zn were determined by inductively-coupled plasma atomic emission spectrometry using 40 mg L^−1^ yttrium as an internal standard in all samples and standard solutions.

### Soil analysis

Total soil metals were measured by atomic absorption spectrometry after digestion with boiling HNO_3_. Exchangeable Ca, Mg, and Ni were obtained by extracting 5 g air-dried soil with 50 mL 1.0 M ammonium acetate at pH 7, soil texture by pipette method, and organic matter by combustion. The Bray-2 method was used to estimate phytoavailable P. The DTPA-extraction used 5 g soil per 50 mL standard DTPA extractant rather than the usual 10 g per 20 mL because of the high metals levels in this and other Ni-rich soils studied in our laboratory.

## Results

**Figure 2:**
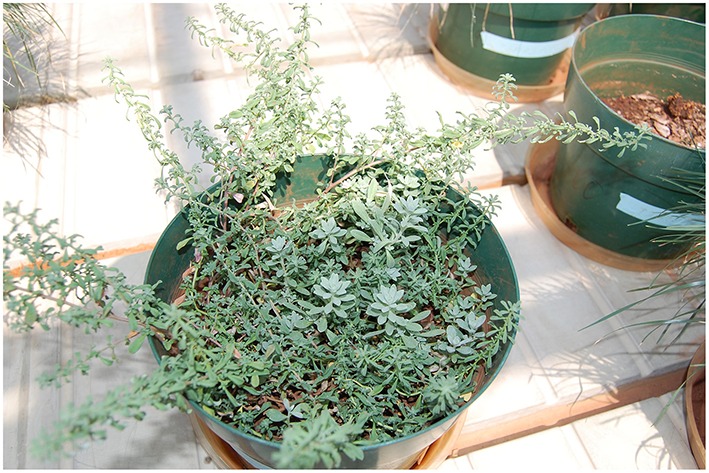
***A. murale* (most of the plant material in pot, with long stems, and darker oblong leaves) and *A. montanum (*a few plants in center with larger, lighter leaves in a rosette pattern) co-cropped in the fertilized serpentine soil**. *A. montanum* grew less vigorously than *A. murale* in the fertilized serpentine soil, and was outcompeted and shaded after 4–5 weeks.

**Figure 3:**
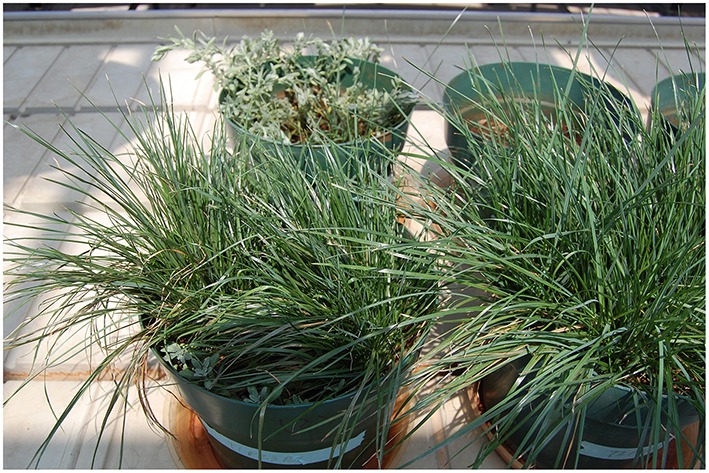
***Lolium perenne and A. montanum* co-cropped in the fertilized serpentine soil**. The nonhyperacumulator *Alyssum* species was not able to compete with the grass.

Yields and dry weight metal concentrations are reported in Table 2, and examples of co-cropped healthy plants in the serpentine soil (**A**) are given in Figures 2, 3. All results were statistically analyzed by ANOVA with SAS. None of the *Alyssum* transplants survived in compost-amended soil **B**. The compost evidently contained pathogen(s) that both species were susceptible to, and it also kept the soil damp longer between waterings. Normally this is desirable in pot studies, however *A. murale* in particular is adapted to semi-arid conditions, and once established survives with watering once per week or less. The symptoms exhibited were consistent with fungal infection. None of the plants in soil **A** or seedlings in Promix were affected.

**Table 2:** **Half-pot dry weight yield and element concentrations of all treatments that survived**.

**Species**	**Treatment**	**Yield g ½ pot^−1^**	**Ca G kg^−1^**	**Cu mg kg^−1^**	**Fe mg kg^−1^**	**K g kg^−1^**	**Mg g kg^−1^**	**Mn mg kg^−1^**	**Ni mg kg^−1^**	**P g kg^−1^**	**Zn mg kg^−1^**
*A. murale*	A1 alone	7.5±3.2	13.6±0.6	2.35±0.42	336±260	14.4±1.2	2.09±0.28	225±150	3320±330	2.92±0.37	27.9±7.6
	A4 + mon	10.3±5.1	13.1±2.4	2.31±0.27	285±240	15.4±1.1	2.08±0.75	202±19	4030±1170	3.32±0.57	20.7±1.4
	A5 + peren	7.87±0.75	12.4±1.3	2.01±0.51	89.3±40	14.1±1.9	2.41±0.62	145±32	3640±830	2.50±0.54	18.1±6.4
*A. montanum*	A2 alone	3.60±0.4	24.1±2.4	1.76±1.1	589±350	19.9±2.6	7.50±1.1	47±10	50±16	3.54±0.55	14.1±2.1
	A4 + mur^*^	1.33	22.0	1.20	342	22.2	6.76	64	57.5	3.54	13.5
	A6 + peren	0.71±0.30	26.0±1.5	2.00±0.37	1689±80	25.9±6.2	9.32±1.3	54.7±21	71.3±14	3.87±0.53	16.8±2.7
*L. perenne*	A3 alone	2.7±0.7	4.37±0.3	6.46±0.06	179±20	25.7±2.5	4.46±0.51	110±28	50.7±4.9	3.95±0.46	29.6±1.5
	A5 + mur	1.75±1.2	4.71±0.84	5.93±0.86	231±120	26.3±5.1	4.78±0.67	202±15	39.7±28	2.96±0.56	28.3±3.2
	A6 + mon	4.01±0.95	4.10±0.62	5.49±1.6	352±350	20.5±6.9	4.38±0.77	102±28	45.7±2.3	2.79±0.34	24.4±3.9
	B3 alone	3.1±1.7	2.45±0.35	8.79±0.92	60.0±00	37.1±5.6	3.49±0.09	52.7±8.1	19.3±4.0	4.00±0.74	82.5±15
	B5 + mur	8.61±2.8	2.25±0.65	10.1±0.97	62.0±27	41.8±11	3.24±0.31	46±8.5	23.3±2.5	3.61±0.70	77.1±5.2
	B6 + mon	7.20±0.79	2.81±0.38	11.1±0.89	96.7±31	46.9±7.4	3.43±0.18	53.3±6.0	19±2.0	3.71±0.32	80.5±2.0

*Mean and s.d. of three replicates except where noted. Soil A: 100% Brockman variant serpentine; soil B Brockman serpentine soil with 10 dw% manure compost mixed in. Plants designated “alone” grown in monoculture; other treatments were mixed culture as listed. Only L. perenne survived in the manure compost amended soil*.

* *only two replicates survived*.

**Figure 4:**
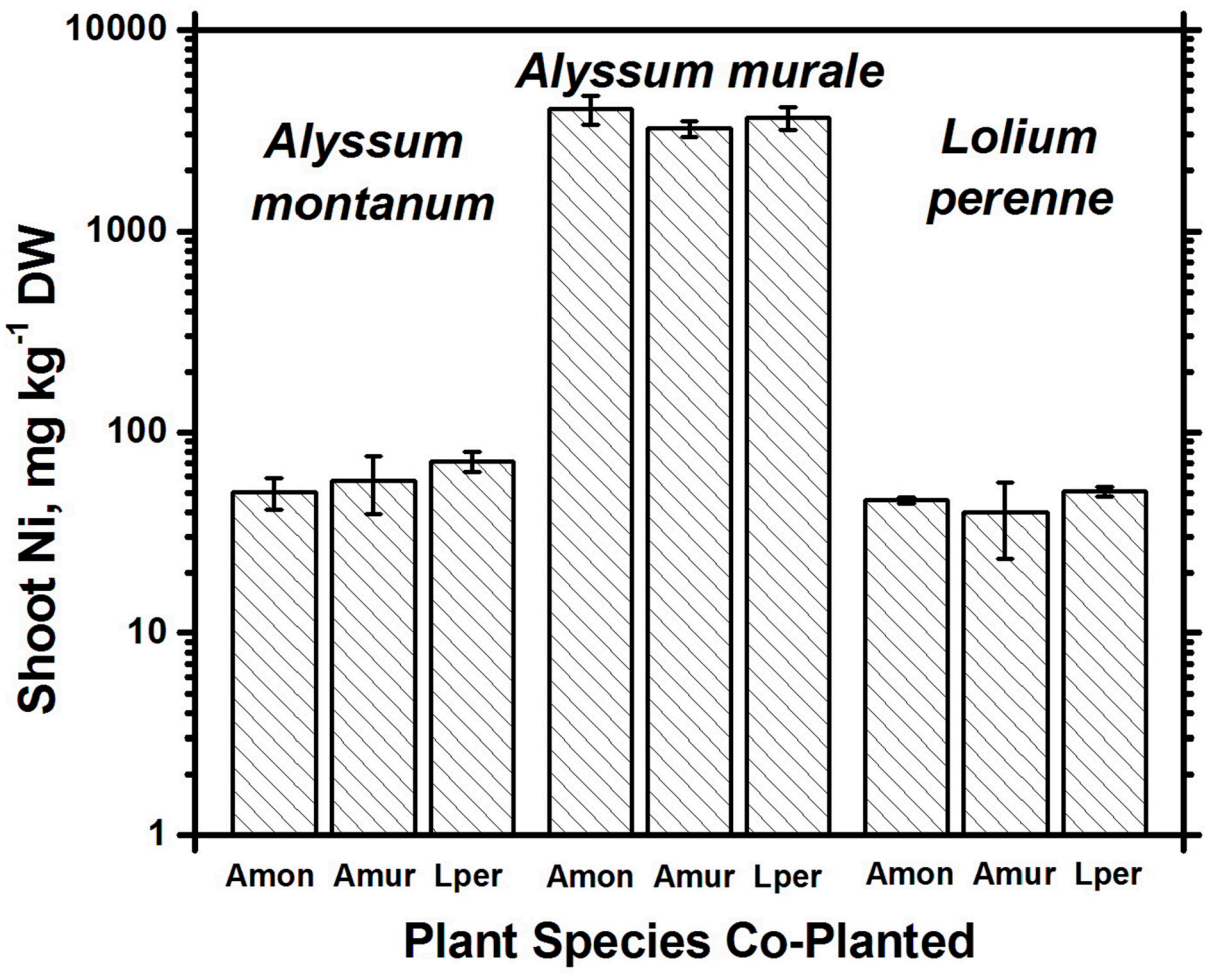
**Ni concentrations for species in monoculture and co-cropped**. Nickel levels were significantly greater in the hyperacccumulator *Alyssum murale* (*p* < 0.001) but did not differ significantly within a given species as a function of co-cropping. Error bars means ± Standard Error.

**Figure 5:**
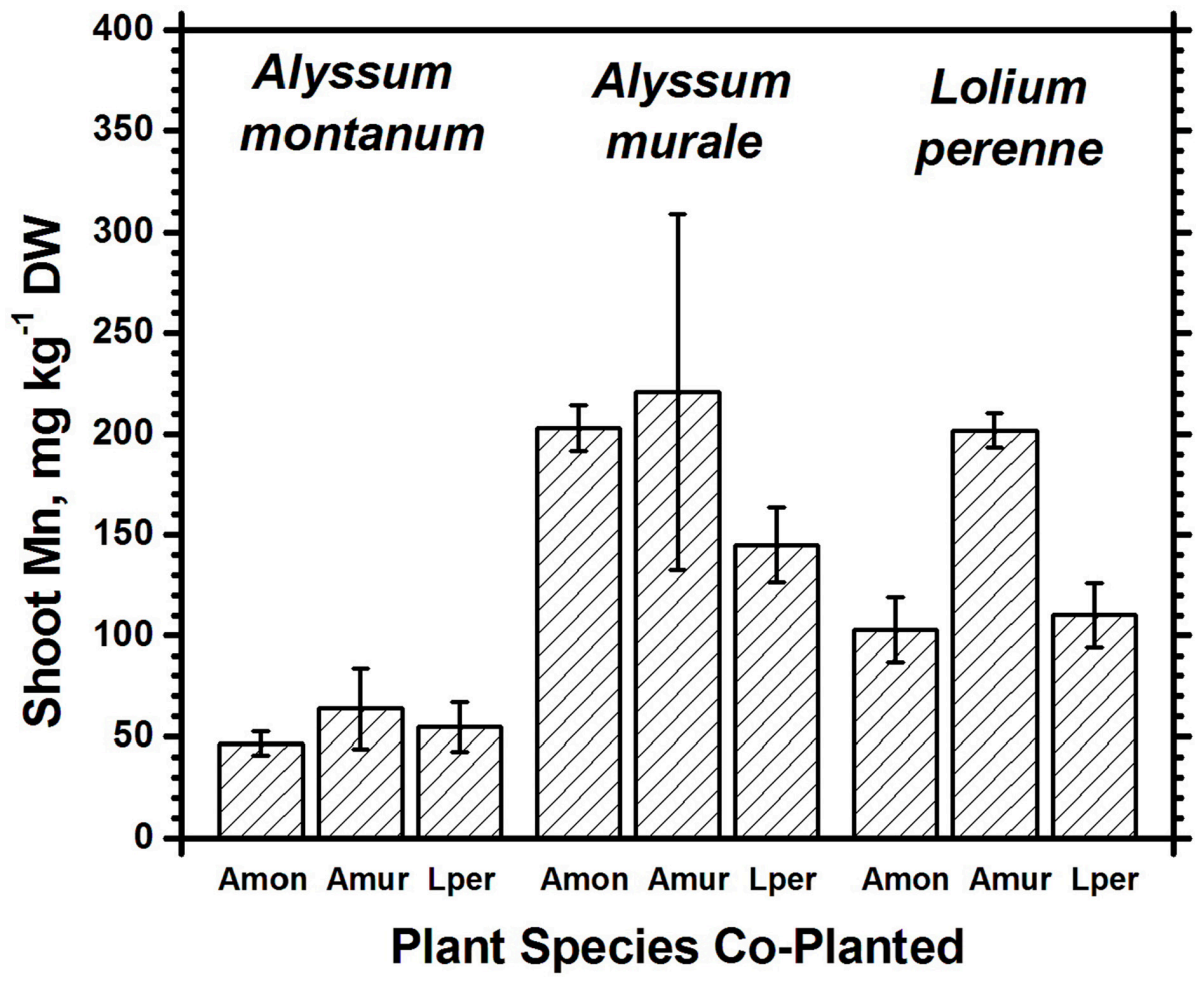
**Mn concentrations for species in monoculture and co-cropped**. Mn levels were significantly greater in *A. murale* than *A. montanum*. Mn was increased in *L. perenne* with *A. murale* co-cropping. Error bars means ± Standard Error.

In soil **A**, *A. murale* shoots contained approximately 3600 mg kg^−1^ Ni which did not differ significantly with co-cropping (Figure 4). Overall Ni and Mn concentrations were significantly higher in *A. murale* than *A. montanum* or *L. perenne* (Figures 4, 5). However, *A. murale* Fe concentrations were significantly reduced (*p* < 0.05) by co-cropping with ryegrass, and Mn was somewhat reduced (*p* < 0.4) but half-pot yield was equivalent. In general Fe concentrations were unreliable in *A. montanum* due to contamination with Fe^3+^ oxide staining deep within the leaves, coupled with only a small amount of plant material available for analysis, but this does not affect the relationship between Ni and Mn in *A. murale* vs. *A. montanum*. Although Fe is much higher in *A. montanum* than *A. murale*, both Ni and Mn are significantly lower (*p* < 0.01), a result that cannot be due to soil contamination.

**Figure 6:**
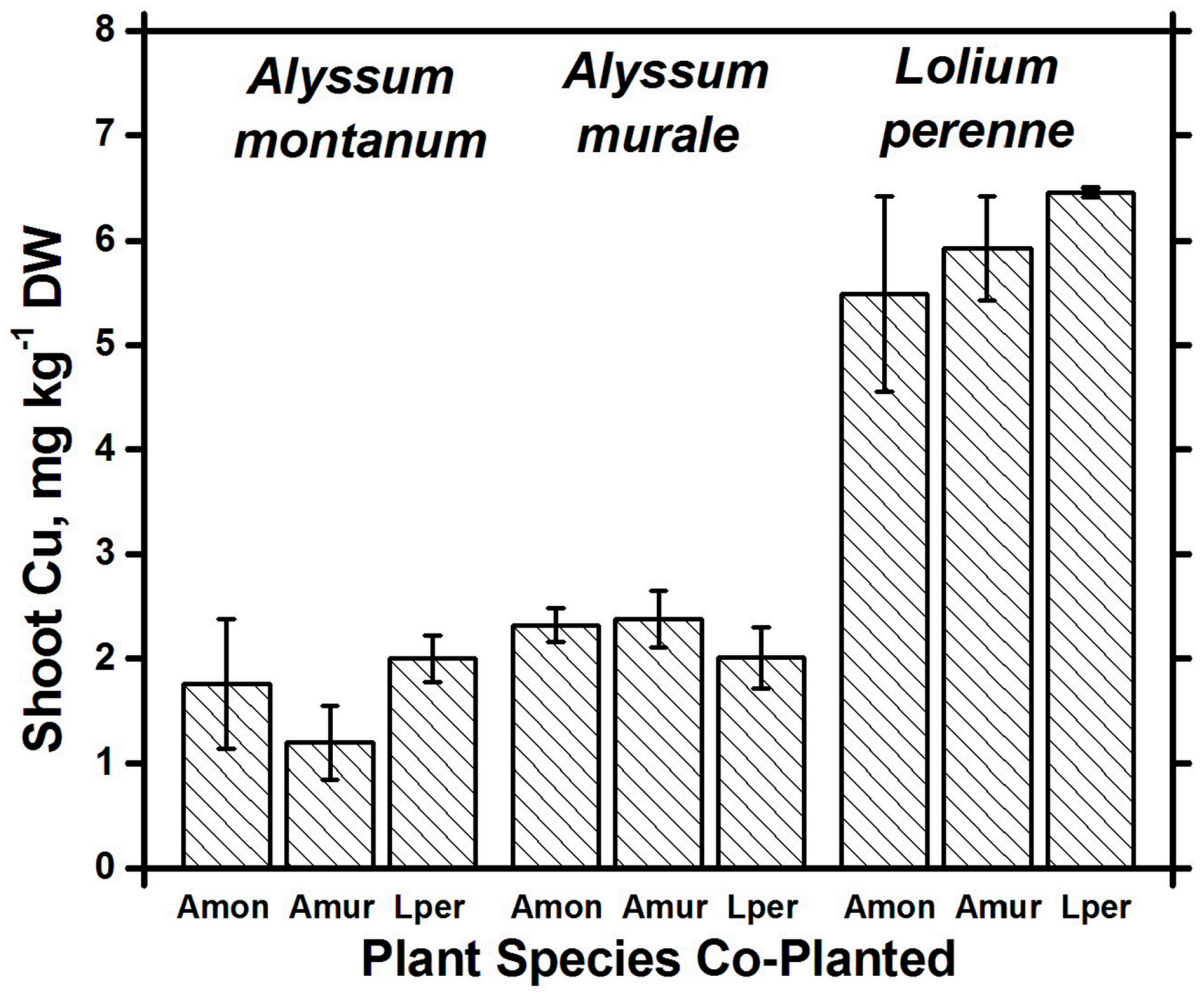
**Cu concentrations for species in monoculture and co-cropped**. Copper levels were significantly greater in *Lolium perenne* compared to both *Alyssum* species (*p* < 0.001). With the exception of a slight Cu reduction in *A. montanum* co-cropped with *A. murale*, Cu concentrations did not differ significantly within a given species as a function of co-cropping. Error bars means ± Standard Error.

Nickel concentrations in *A. montanum* and *L. perenne* remained relatively constant and below 75 mg kg^−1^ in for **A2** through **A6**. However, there was increased variability of the Ni concentration in both *A. murale* or *L. perenne* with *A. murale* intercropping, and intercropping with *A. murale* significantly increased Mn in *L. perenne* (Figure 5). Calcium concentrations were three to six times greater in *Alyssum* species as compared to *L. perenne* due to the high Ca in *Alyssum* leaf trichomes (Table 2; Figure 1). However, Cu concentrations were consistently greater in *L. perenne* than *Alyssum* (Figure 6).

In soil **A**, *A. montanum* could not compete with either *A. murale* or ryegrass and was nearly killed by co-cropping, with 10-fold yield reductions (Figures 3, 4). Due to its poor growth, *A. montanum* did not significantly affect the growth or metal concentrations of *A. murale* or *L. perenne*. However, there was a significant ryegrass yield reduction with *A. murale* co-cropping. Ryegrass yield was increased when co-cropped with *A. montanum* because it thoroughly out-competed *A. montanum* with only half the plants.

Because *Alyssum* did not survive in the treatments with compost, all three ryegrass planting schemes grew ryegrass only. Essentially there were nine replicates for **B3**, all of which had Ni concentrations that did not differ significantly from one another, but did differ from **A3** and **A5**, as expected due to the high levels of Ni in the serpentine soil (Figure 4). There was a trend for increased yield with compost but it was not significant. Calcium, Fe, Mg, and Ni concentrations in ryegrass were reduced with compost and Cu and Zn concentrations were increased. Ni concentrations in ryegrass were about twice as high in treatment **A** compared to **B**, while the reverse was true for Cu and Zn.

## Discussion

Our results indicate there is no value with respect to phytomining or phytoextraction in co-cropping *A. murale* with *L. perenne*. Neither yield nor Ni uptake was improved; the ryegrass shoots only interfered with the growth of *A. murale*. The average full-pot yield for *A. murale* grown alone was 15.0 ± 6.3 g, therefore phytoextraction could be doubled just with *Alyssum* monoculture. Further, given larger pot sizes or field growth, *A. murale* and *A. corsicum* develop extensive root systems than can increase shoot Ni concentration up to five times that achieved in 1 kg pots (Baklanov et al., 2015; Bani et al., 2015a). Therefore, root growth interference from the equally extensive *L. perenne* root system is almost certainly a negative factor with respect to maximizing Ni phytoextraction. Co-cropping *Lupinus albus* and *A. murale* in natural serpentine soils showed similar results in a study investigating whether co-cropping with a nitrogen-fixing plant could improve overall *A. murale* Ni phytoextraction (Jiang et al., 2015). Without supplemental P fertilization, 90% of the biomass in the pots was *L. albus*. With P fertilization *A. murale* increased to 39%, however Ni accumulation in the shoots was significantly reduced compared to monocropping. Overall Ni phytoextraction was maximized in the monocrop with P fertilization.

Co-cropping with ryegrass somewhat reduced Fe and Mn concentrations in *A. murale* but not to the extent of either increasing Ni uptake or affecting plant nutrition, so the result, while interesting, has a neutral effect on phytoextraction in this soil. The increased variability of the Ni concentration in both *A. murale* and *L. perenne*, and increased Mn in *L. perenne* with *A. murale* co-cropping may reflect increased rhizosphere mobilization of Ni and Mn by *A. murale* but in this experiment it did not translate to any tangible benefit. The hypothesized increase in Ni accumulation in response to phytosiderophores secreted by co-cropped grasses clearly did not occur. Our data do not support increased mobilization of Mn by a phytosiderophore mechanism either, but the converse: mobilization of Mn by the *Alyssum* hyperaccumulator species significantly increased Mn levels in the grass.

*A. montanum* could not compete with either *A. murale* or ryegrass and was nearly killed by co-cropping. In field growth it would be unlikely to survive. In contrast to results with *Noccaea* (Whiting et al., 2001b), there would be no value in utilizing an *Alyssum* hyperaccumulator to improve the growth of a non-hyperaccumulator; *A. montanum* yield was strongly reduced by co-cropping, yet it grew well alone in the fertilized serpentine soil.

*A murale* and *A. montanum* accumulated about 13 and 24 g Ca kg^−1^ respectively, consistent with all previous observations in which CaCO_3_ nodules cover the surface of the trichomes (Krämer et al., 1997; Psaras et al., 2000; Küpper et al., 2001; Kerkeb and Krämer, 2003; Marmiroli et al., 2004; Broadhurst et al., 2004a,b; Broadhurst et al., 2009; McNear et al., 2005; Asemaneh et al., 2006; Tappero et al., 2007). Calcium fertilization was necessary for *L. perenne* growth in this experiment, however *Alyssum* hyperaccumulator species native to typically low Ca, low Ca:Mg ratio serpentine soils nonetheless accumulate Ca in the absence of fertilization. *A. montanum* is not a Ni hyperaccumulator but had twice the Ca of *A. murale*, but only half the Mn.

Both Ni and Mn concentrations were significantly higher in *A. murale* than *A. montanum* or *L. perenne*. The high variability in the *A. murale* Mn concentration is typical of the species, which in natural serpentine soils is observed to hyperaccumulate Mn only in some leaves on a given plant. If Mn soil levels are exceedingly high without addition of Ni, Mn is not hyperaccumulated throughout the plant and instead becomes phytotoxic (Broadhurst et al., 2004b, 2009; Tappero et al., 2007). Ni hyperaccumulators are very specific to Ni and to a lesser extent Mn and Co, and do not non-selectively accumulate/hyperaccumulate other transition metals such as Fe, Cr, or Cu. In the case of Cu, despite 3600 mg kg^−1^ Ni accumulation, *A. murale* Cu concentrations were only 2 mg kg^−1^, far below *L. perenne*, which accumulated typical foliar Cu levels for ryegrass. These observations support a specific relationship between Mn accumulation and Ni hyperaccumulation (Broadhurst et al., 2009; Ghaderian et al., 2015) rather than a general situation for *Alyssum* species where Mn uptake and storage is related to enhanced Ca uptake to synthesize the unique trichome tissues (McNear and Kupper, 2013).

Although the compost utilized was a standard, mature aged product from USDA Beltsville it was very detrimental to *Alyssum* growth, most likely due to pathogenic fungi. We have repeatedly observed fungal infections in *Alyssum* species grown in humid summer greenhouse conditions. Although we grew the plants in the late winter/spring season and in a majority of their native soil, they were nonetheless unable to survive transplant to the manure compost amended soil. Seeding in the pot was tried but the germination rate of *A. montanum* was below 40% and seedlings that did come up were very weak. Several plants were transplanted to soil **B** and grown outdoors but also succumbed with the same disease pattern. However, both *Alyssum* species grew very well alone in the fertilized serpentine soil with no evidence of disease or phytotoxicity.

In a similar recent study, Álvarez-López et al. (2016) grew the hyperaccumulators *A. serpyllifolium ssp. lusitanicum, A. serpyllifolium ssp. malacitanum, A. pintodasilvae*, and *A. bertolonii*. in their native serpentine soil with 2.5, 5, and 10 wt% commercial municipal solid waste compost added. These species grow slowly so did not achieve the large shrub size that *A. murale* can in one season. The lower levels of compost addition significantly increased yield but no further benefits were achieved with 10%. All levels of compost addition reduced extractable Ni; at the 10% level the reduction was 11-fold. Overall yield was lower without compost but with inorganic NPK fertilization, however NPK addition did not affect Ni accumulation. In an Albanian field trial with ultramafic Vertisols, Bani et al. (2015b) found *A. murale* yield was increased 10-fold with 120 kg NPK and 77 kg Ca ha^−1^ plus monocot herbicide to control Graminaceae—as opposed to encouraging co-cropping. These agronomic practices increased Ni phytoextraction yield from 2.0 to 29.5 kg ha^−1^. Thus, in a long-term field Ni phytoextraction or phytomining situation, standard inorganic fertilization may be both adequate and preferable. If a compost source is utilized, it should be tested with every species/ecotype used in the field program prior to application. Another factor to consider is a possible negative effect of compost biota on serpentine-endemic rhizobacteria which can act to facilitate Ni uptake. The two bacteria shown to strongly increase Ni accumulation in *A. murale* (*M. arabinogalactanolyticum* and *M. oxydans*) were isolated from the Oregon soil that we utilized in this study (Abou-Shanab et al., 2003, 2007), thus were potentially present in each pot. They may not have thrived in the compost-amended soil just as the *Alyssum* species did not, however rhizobiome interactions cannot explain our results.

Ryegrass growth was not negatively affected by the compost and the Ni concentration was significantly reduced without inducing Fe or Mn deficiency. With fertilization and adequate water ryegrass grew reasonably well on the serpentine soil; adding compost would be a significant benefit to retain soil moisture and improve root growth. Ryegrass yield may have increased in treatment **B** if grass was cut one or two times during the experiment. This was not done because in a field intercropping situation *A. murale* would need to grow as long as the season permits in order to maximize Ni phytoextraction, and it would not be practicable to selectively cut the ryegrass. Similarly, regular light irrigation and cool, relatively humid conditions to maximize *L. perenne* yield would not be practicable since *A. murale* grows better with infrequent but thorough waterings and relatively hot, sunny, low humidity conditions. In commercial phytomining of Ni, weed control to prevent grasses would normally be practiced to limit competition for water and nutrients (Bani et al., 2015b).

Overall, tilling soil to maximize root penetration, adequate inorganic fertilization and appropriate plant densities are more important for developing efficient phytoremediation and phytomining approaches with *Alyssum* Ni hyperaccumulator species than organic soil amendments or co-cropping.

## Author contributions

CB Principal Experimentalist, analyzed data, and wrote ms. RC Research Group Leader, involved in all experimentation in the laboratory. Co-designed experiment and co-wrote ms.

## Funding

RC is a Federal Employee, US Department of Agriculture Agricultural Research Service.

CB is posted at US Department of Agriculture Agricultural Research Service and is a State of Maryland Employee.

### Conflict of Interest Statement

The authors declare that the research was conducted in the absence of any commercial or financial relationships that could be construed as a potential conflict of interest.
